# Polysaccharides from *Volvariella volvacea* Mushroom: Extraction, Biological Activities and Cosmetic Efficacy

**DOI:** 10.3390/jof8060572

**Published:** 2022-05-26

**Authors:** Sarita Sangthong, Punyawatt Pintathong, Patcharee Pongsua, Areeya Jirarat, Phanuphong Chaiwut

**Affiliations:** 1 School of Cosmetic Science, Mae Fah Luang University, Chiang Rai 57100, Thailand; sarita.san@mfu.ac.th (S.S.); punyawatt.pin@mfu.ac.th (P.P.); 2 Green Cosmetic Technology Research Group, Mae Fah Luang University, Chiang Rai 57100, Thailand; patcharee.pon@mfu.ac.th (P.P.); areeyajirarat@gmail.com (A.J.)

**Keywords:** anti wrinkle, efficacy, cosmetics, moisturizing, polysaccharides, *Volvariella volvacea*, whitening

## Abstract

Polysaccharides from *Volvariella volvacea* (VVP) were investigated for their cosmetic-related activities and in vivo efficacy for use as a multifunctional active cosmetic ingredient. Three different polysaccharide extraction methods, including hot water shaking (HS), microwave-assisted (MA) and ultrasonic-assisted (UA), were used. Extractable yield, polysaccharide contents and biological activities, including antioxidant, anti-tyrosinase and anti-elastase activities, were compared. The polysaccharides from HS provided the highest extraction yield (15.58 ± 0.96% *w*/*w*) and the highest beta-glucan content (18.80 ± 0.81% *w*/*w*). The HS polysaccharides also possessed the highest inhibitory effects toward lipid peroxidation (IC_50_ of 0.0378 mg/mL), tyrosinase (51.46 mg KAE/g), and elastase (604.21 ± 73.66 mg EGCG/g). The cytotoxicity of the VVP was determined for safe use. A cosmetic gel cream containing VVP was developed and 0.2% VVP formulation was observed to be the most stable in color. UV protection factors, skin irritation by single patch test, and in vivo efficacy, including skin moisturization, anti-wrinkle and whitening, were measured. The VVP showed no cytotoxicity against human dermal skin fibroblast. The gel cream containing VVP provided less sun protection factor; however, it significantly exhibited the skin benefits of increasing moisture, gross elasticity, net elasticity, and skin firmness. Improvements to skin roughness, scaliness, wrinkles and in melanin content were also depicted gradually along 8 weeks. *V. volvacea,* therefore, could be a good source for polysaccharides being used as a moisturizing, anti-wrinkle, and whitening agent in cosmetic preparations.

## 1. Introduction

Edible mushrooms are considered nutritious foods due to their chemical profile, being high in carbohydrates, proteins, dietary fiber, vital vitamins, and minerals, but low in calories and fats [1]. In recent literature it was reported that edible mushrooms, are a source of proteins and peptides with antifungal activity. Ribotoxin-like proteins (RL-Ps), which are enzymes possessing several interesting features, including antifungal activity, were found in various edible mushrooms [2]. Several peptides from edible mushrooms with numerous biotechnological activities, including antifungal activity, were also reported [3]. The biological activities of mushrooms received considerable interest due to a variety of health benefits, including immunomodulation, anti-cancer activity, cardiovascular therapies, and antiviral and antibacterial actions [4]. Polysaccharides are one category of chemicals found in mushrooms; many researchers improved their investigations on the efficient extraction and functional properties of these for health benefits. Generally, bioactive polysaccharides from mushrooms consist of *β*-glucan that occurs in terms of *β*-(1–3)- and *β*-(1–6)-linked glucose groups with different patterns and degrees of branching. The *β*-glucan acts as an immunomodulator that enhances macrophage activity, which relates to collagen biosynthesis in wound healing and wrinkle reduction [5]. In addition, β-glucan has the ability to scavenge free radicals, reduce infection induce strong tissue regeneration in animal and human models, and is anti-inflammatory [6,7]. Mushroom polysaccharides, including *Velutipes* mushroom polysaccharides [8] and Tremella [9], were reported for being natural biological macromolecules with the presence of a large number of hydrophilic hydroxyl groups, having strong hygroscopicity and good film-forming properties in moisturizer products. Natural polysaccharides suppress dryness of the skin and prevent wrinkles in anti-aging products [10].

*Volvariella volvacea,* or straw mushroom, is an edible mushroom that is widely cultivated in East and Southeast Asia. In general, the dominant characteristic is a white round shape that slowly grows and ruptures. The fruiting body comprises stalks growing above ground, with mushroom caps then spreading out like an umbrella, gray in color, and gills located under the cap [11]. The fruiting body of *V. volvacea* is a rich source of enzymes which act as inhibitors of protein synthesis named ribosome inactivating proteins [12]. *V. volvacea* polysaccharides (VVP) are interesting in terms of their biological activities. Beta-glucan was found to be a major polysaccharide in *V. volvacea.* The backbone chain of VVP is β-(1→3)-linked-D-glucose residues, one out of five or six being substituted at O-6 with a single glucosyl or β-(1→6)-linked diglucosyl group [13]. In a previous study, aqueous extracts of *V. volvacea* were represented in in vitro collagen biosynthesis stimulating activities on the human skin fibroblast cell line, and antioxidant activities [14]. *V. volvacea* has pharmaceutical value due to its antitumor polysaccharides, immunosuppressive proteins, and immunomodulatory lectins [11]. VVP can, therefore, be considered to be a promising potential cosmetic ingredient; however, evidence relating to extraction of the VVP and its cosmetic biological activity is still required. Polysaccharides have been extensively extracted by hot water treatment. In the last decade, novel green methods such as microwave-assisted and ultrasonic-assisted extractions have been introduced for polysaccharide separation because of their lower time and energy consumption.

This study, therefore, aimed to extract VVP using different extraction methods and compare its biological activities. The obtained VVP was investigated for its potential use as an active cosmetic ingredient both in vitro and in vivo and for which the application of VVP in the cosmetic field has not yet been reported. The benefit obtained from the study would be the use of *V. volvacea* as a potential source of water-soluble polysaccharides because of its highly effective natural active ingredient which is safe for cosmetic applications.

## 2. Materials and Methods

### 2.1. Materials

Fresh *Volvariella volvacea* was purchased locally from Phan District, Chiang Rai, Thailand. Its voucher number, which was collected at Mae Fah Luang University, was MFUMR004. Trolox ((±)-6-hydroxy-2,5,7,8-tetramethylchromane-2-carboxylic acid), kojic acid, epigallocatechin gallate (EGCG), DPPH (2,2-diphenyl-1-picrylhydrazyl), ABTS (2,2′-azino-bis (3-ethylbenzothiazoline-6-sulfonic acid), TPTZ (2,4,6-Tris(2-pyridyl)-s-triazine), L-DOPA (3,4 dihydroxy-L-phenylalanine), mushroom tyrosinase (EC 1.14.18.1), porcine pancreatic elastase (EC 3.4.21.36), and N-Succ-(Ala3-p-nitroanilide) were purchased from Sigma-Aldrich (St. Louis, MO, USA). Minimum Essential Medium (MEM), Dulbecco’s Modified Eagle’s Medium (DMEM), fetal bovine serum (FBS), L-glutamine, penicillin, and streptomycin were purchased from ThermoFisher Scientific (Waltham, MA, USA).

### 2.2. Sample Preparation

The mushrooms were washed with tap water and air dried before drying in a tray dryer at 55 °C until the weight was constant, its moisture content being less than 10% *w*/*w*. The dried samples were pulverized into 250 µm size particles and kept at −20 °C.

### 2.3. Chemical Composition Analysis

Chemical compositions of *V. volvacea* mushrooms including moisture, ash, crude protein, crude fat, crude fiber, and total carbohydrate were determined according to the Association of Official Analytical Chemist (AOAC) methods [15]. For moisture content, powder samples were dried in an oven at 105 °C overnight until the weights were constant. The ash content was determined by incineration at 500 °C for 6 h or until the color of the sample powder was white. The crude protein was determined by the micro-Kjeldhal method. The sample was digested with sulfuric acid in the Kjeltec digestion apparatus (1007 Digestion Unit, Tecator, Sweden). The total protein content was estimated using a nitrogen factor of 4.38 [16]. The crude fat was determined by treating the powder sample with petroleum ether as a solvent in the Soxhlet apparatus. For the crude fiber, Celite 545 dissolved in sulfuric acid was used as a filter agent, then the sample was boiled with sodium hydroxide. The residue in the crucible was dried in the oven at 105 °C until constant weight and placed in the muffin furnace at 500 °C. The total carbohydrate was calculated by 100-(%moisture—%ash—%protein—%fat). All analyses were done in triplicate and averaged.

### 2.4. Polysaccharide Extraction

The VVP was extracted by modifications from previous reports which stated the condition providing the highest yield of polysaccharides extracted by hot water shaking (HS) [17], ultrasonic-assisted (UA) [18] and microwave-assisted (MA) extraction [19].

#### 2.4.1. Polysaccharide Extraction Using Hot Water Shaking (HS)

The method was modified from that reported by Yap and Ng [17]. The powdered sample was mixed with hot deionized water (ratio 1:100 *w*/*v*) and stirred for 10 min. The mixture was then shaken at 95 °C for 5 h. The aqueous extract was separated by centrifugation at 9000 rpm and the supernatant was collected. The solution was precipitated with 95% ethanol (ratio 1:5 *v*/*v*) at 4 °C for 12 h. The precipitate was collected by centrifugation, and then lyophilization.

#### 2.4.2. Polysaccharide Extraction Using Ultrasonic Assistance (UA)

Polysaccharides from the *V. volvacea* were extracted by ultrasonication which was modified from a previous method [18]. The powdered sample was mixed with hot deionized water (ratio 1:100 *w*/*v*) and stirred for 10 min. The polysaccharides were then extracted using sonication at 95 °C for 5 h. The aqueous extract was separated by centrifugation at 9000 rpm and the supernatant was collected. The solution was precipitated with 95% ethanol (ratio 1:5 *v*/*v*) at 4 °C for 12 h. The precipitate was collected by centrifugation, and then lyophilization.

#### 2.4.3. Polysaccharide Extraction Using Microwave Assistance (MA)

The polysaccharide was extracted by mixing a powdered sample with hot deionized water (ratio 1:100 *w*/*v*) and stirring for 10 min. Extraction was then performed using microwave apparatus at 860 W for 25 min [19]. Aqueous extracts were separated by centrifugation at 9000 rpm and the supernatant was collected for protein elimination with Sevag reagent. The solution was precipitated with 95% ethanol (ratio 1:5 *v*/*v*) at 4 °C for 12 h. The precipitate was collected by centrifugation, and then lyophilization.

### 2.5. Determination of Polysaccharide Contents

Polysaccharide contents were determined by phenol–sulfuric acid assay as described by Dubois et al. [20] and glucose was used as standard. The proper volume of diluted sample was added and adjusted to 0.50 mL with deionized water. An amount of 0.50 mL of 5% phenol solution was then added, followed by 2.50 mL of sulfuric acid (H_2_SO_4_). The mixture was vortexed and incubated at 50 °C for 20 min, then stored at room temperature to cool down the mixture. The reaction measured the absorbance at 490 nm against the blank with absence of sample, using a UV-Vis spectrophotometer. The result was expressed as gram glucose equivalent per gram of freeze-dried sample (g GE/g FD).

### 2.6. Determination of Beta-Glucan Content

The β-glucan content of VVP was determined using a mushroom and yeast specific β-glucan kit (K-YBGL, Megazyme, Bray, Ireland), according to method reported by Bak et al. [21]. The enzyme kit contained exo-1,3-β-glucanase plus β-glucosidase, amyloglucosidase plus invertase, reagent enzymes (glucose oxidase plus peroxidase, and 4-aminoantipyrine), D-glucose standard solution, and control fungal β-glucan preparation. Measurement of total glucan content was conducted by hydrolyzing the mushroom samples with 37% hydrochloric acid (*v/v*) at 30 °C for 45 min followed by an additional heating period at 100 °C for 2 h. Subsequently, the sample was neutralized with 2.0 M potassium hydroxide. Glucose hydrolysis was performed using a mixture of exo-1,3-β-glucanase and β-glucosidase in 200 mM sodium acetate buffer pH 5.0 at 40 °C for 1 h. The absorbance of the resulting complex color was measured at 510 nm using a spectrophotometer. On the other hand, the α-glucan content was determined using the method described above following enzymatic hydrolysis with amyloglucosidase plus invertase. The β-glucan content was calculated by subtracting the α-glucan content from the total glucan content. Glucan content was expressed as a percentage (%*w*/*w*) of mushroom dry weight.

### 2.7. Determination of Antioxidant Capacity

#### 2.7.1. ABTS Radical Scavenging Capacity Assay

The decolorizing of 2,2′-azino-bis (3-ethylbenzothiazoline-6-sulphonic acid) cation radical scavenging capacity of the extract was determined. For ABTS, radical scavenging capacity was determined by a spectrophotometric method according to the procedure reported by Thaipong et al. [22] with minor modifications. The ABTS^●^ (7.4 mM) solution and potassium persulfate (2.45 mM) solution were mixed together and kept in dark ambient conditions for 12–16 h prior to use for ABTS^●+^ generation. The stock ABTS^●+^ was mixed with phosphate buffer (50 mM) pH 7.4 before the test. The proper volume of diluted sample was added with 50 mM phosphate buffer pH 7.4 into 1.0 mL. An amount of 2.0 mL ABTS^●+^ reagent was then added, vortexed, and incubated at dark ambient condition for 30 min. The absorbance of the reaction mixture was measured at 734 nm and decrement of absorbance was calculated against the control of the stable cation radical of ABTS as per the equation below:

ABTS radical scavenging capacity (%) = ((A_734 control_ − A_734 sample_)/A_734 control_) × 100 (1)

where, A_734 control_ = the absorbance of the control (reaction without antioxidant); and A_734 sample_ = the absorbance of the sample extract or trolox standard.

The trolox standard curve was prepared by plotting the percentage of inhibition versus trolox concentration. The result was expressed as milligram trolox equivalent antioxidant capacity per gram sample (mg TEAC/g).

#### 2.7.2. Ferric-Reducing Antioxidant Power (FRAP) Assay

Ferric-reducing antioxidant power was determined according to the method reported by Thaipong et al. [22] and Benzie and Strain [23] with modification. The complex of the reduced form of ferrous, Fe (II) with 2,4,6-Tri(2-pyridyl)-s-triazine (TPTZ) was measured. The FRAP reagent was prepared by mixing the FeCl_3_ (20 mM), TPTZ (10 mM), and acetate buffer (0.3 M) pH 3.6 (ratio 1:1:10 *v*/*v*) and kept in an amber bottle. The proper volume of diluted sample was added with the adjusting of 0.3 M acetate buffer pH 3.6 into 1.0 mL. An amount of 2.0 mL FRAP reagent was then added, vortexed, and incubated at 37 °C for 30 min. The absorbance was measured by UV-Vis spectrophotometer at 593 nm against the blank with the absence of sample. The antioxidant capacity was calculated into milligram trolox equivalent antioxidant capacity per gram sample (mg TEAC/g).

#### 2.7.3. Lipid Peroxidation Inhibition Activity Assay

The lipid peroxidation inhibitory activity was determined according to the method reported by Zhang and Kirkham [24] with modification. The linoleic emulsion (0.5 mL) and 0.1 mL ferrous sulphate (4 mM) were added into a screw test tube. The proper volume of diluted sample was added with the adjusting of Tris-buffer pH 7.5 into 0.6 mL. An amount of 0.1 mL ascorbic acid was then added, vortexed, and incubated at 37 °C for 10 min. An amount of 1.0 mL TBA-TCA solution was added and incubated at 95 °C for 5 min. The mixture was then placed in an ice bath for 5 min to stop the reaction. The emulsion reaction was centrifuged at 7000 rpm for 5 min, then, the absorbance of the supernatant was measured at 532 nm using a UV-Vis spectrophotometer. The blank was set without the adding initiator (Fe_2_SO_4_) and the control was absence of sample. The lipid peroxidation inhibitory activity was calculated into percentage of inhibition as per the equation below:

Lipid peroxidation inhibitory activity (%) = ((A_532 control_ − A_532 sample_)/A_532 control_) × 100 (2)

where, A_532 control_ = the absorbance of the control (reaction without antioxidant); and A_532 sample_ = the absorbance of the sample extract or BHT standard.

The BHT standard curve was prepared by plotting the percentage of inhibition versus BHT concentration. The result expressed 50% inhibitory activity (IC_50_; mg/mL).

### 2.8. Determination of Tyrosinase Inhibitory Activity

The mushroom tyrosinase (EC 1.14.18.1) inhibitory activity was determined using L-Dopa as the substrate, according to the method reported by Onar et al. [25]. The mushroom tyrosinase (EC 1.14.18.1) was prepared into 1000 units/mL and was used in 0.02 mL. The proper volume of diluted sample was mixed with 50 mM potassium phosphate buffer pH 6.8 into 0.16 mL. The mixture was incubated at 37 °C for 5 min. A quantity of 0.02 mL 25 mM L-DOPA was then added, and the mixture was incubated at 37 °C for 15 min exactly. The absorbance of the reaction mixture was measured at 475 nm using a microplate reader. The blank was set without adding the tyrosinase enzyme and the control was the absence of a sample. The percentage of tyrosinase inhibition was calculated against the control as per the equation below:

Tyrosinase inhibition (%) = ((A_475 control_ − A_475 sample_)/A_475 control_) × 100 (3)

where, A_475 control_ = the absorbance of the control (reaction without kojic acid); and A_475 sample_ = the absorbance of the sample extract or kojic acid standard.

The kojic acid standard curve was prepared by plotting the percentage of inhibition versus kojic acid concentration. The result was expressed as milligram kojic acid equivalent tyrosinase inhibitory activity per gram sample (mg KAE/g).

### 2.9. Determination of Elastase Inhibitory Activity

The porcine pancreatic elastase (EC 3.4.21.36) inhibitory activity of VVP was evaluated according to the method reported by Lee et al. [26]. The porcine pancreatic elastase inhibitory activity against the substrate of N-Succ-(Ala3-p-nitroanilide) was determined and epigallocatechin gallate (EGGC) was used as the standard substance. The proper volume of the diluted sample was mixed with 0.2 M Tris-hydrochloride buffer pH 8.0 (155 µL) and added into 10 unit/mL porcine pancreatic elastase (2.5 µL). The mixture was incubated at 37 °C for 10 min, then 2.5 µL of the substrate (5 mM) was added and incubated at 37 °C for 20 min exactly. The absorbance of the reaction mixture was measured at 410 nm using a microplate reader. The blank was set without adding porcine pancreatic elastase enzyme and the control was absence of sample. The percentage of elastase inhibition was calculated against the control as per the equation below:

Elastase inhibition (%) = ((A_410 control_ − A_410 sample_)/A_410 control_) × 100 (4)

where, A_410 control_ = the absorbance of the control (reaction without EGCG); and A_410 sample_ = the absorbance of the sample extract or EGCG standard.

The epigallocatechin gallate (EGGC) standard curve was prepared by plotting the percentage of inhibition versus EGGC concentration. The result was expressed as milligram EGCG equivalent elastase inhibitory activity per gram sample (mg EGCGE/g).

### 2.10. UV Absorption Scanning

The VVP absorbability was scanned in the UV region at a wavelength between 200 and 400 nm and a frequency of 1 nm by a UV-Vis spectrophotometer, according to the method reported by Bandasak et al. [27]. The absorbability of VVP was compared to the standard UV absorbance of oxybenzone.

### 2.11. Cytotoxicity Test

#### 2.11.1. Cell Culture

Human dermal skin fibroblast cells were grown in Dulbecco’s Modified Eagle’s Medium (DMEM) supplemented with 10% fetal bovine serum, 2 mM L-glutamine, 100 units/mL penicillin, and 100 µg/mL streptomycin. The cells were incubated in a humidified incubator with an atmosphere of 95% air and 5% CO_2_ at 37 °C.

#### 2.11.2. MTT Cytotoxicity Test

The screening method by MTT assay was adopted from the published standard methods (BS-EN30993-5 and ISO10993-5). Briefly, metabolism component cells were able to metabolize the tetrazolium to formazan. These assays investigated the ability of compounds for inhibition of cell growth which were based on fluorometric detection of viable cells. Ellipticine was used as a positive substance. The color change was measured by a microplate reader. It could be assumed that cells that were metabolically deficient would not survive. The cells were seeded in a 96-well plate at a density of 2000 cells/well and incubated for 48 h. The VVP was added to the cells and incubated for 24 h. The polysaccharides were removed from cell cultures, then, the cells were re-incubated for a further 24 h in fresh medium before the addition of a chromogenic dye, 3-(4,5-dimethylthiazol-2-yl)-2,5-diphenyl tetrazolium bromide (MTT). After 4 h of incubation, the medium in the 96-well plates was removed, and 200 µL of DMSO was added to each well and mixed thoroughly to dissolve the formazan crystals. Absorbance was measured using a microplate reader (molecular device) at 570 nm. The data were analyzed with the SoftMax Program to determine the IC_50_.

### 2.12. Development of Gel Cream Containing VVP

The cream base formula in Table 1 was developed. The order of mixing was performed under the regular mixing method of oil in water emulsion type. The ingredients were weighed separately. The ingredients of part A were weighed and mixed until homogeneous, prior to being added to the other part, and mixed well. In part B, citric acid was dissolved in deionized water. Part B was added into part A and mixed until homogeneous. Emulsion was formed by adding part C into the previous mixture. The emulsion was thoroughly mixed before parts D and E were added, respectively.

The cream base formula was evaluated for stability through four cycles of heating–cooling treatment by measuring pH and viscosity, and color by eye perception. The stable cream base formula was used to prepare cream containing VVP. The gel cream containing VVP was formulated with 3 varying concentrations: 0.2, 0.5, and 1.0% (*w*/*w*).

#### 2.12.1. Stability Test for Gel Cream Containing VVP under Various Conditions

A preliminary stability check, the phase separation after the centrifugation, was performed. The gel cream, 1.0 mL in a micro-tube, was centrifuged at 5000 rpm for 30 min. The separation layer of gel cream was evaluated.

The accelerated condition of the heating–cooling cycle was performed by storing the gel cream, 100 g in a lid-closed glass bottle, initially at 45 °C for 24 h, and then at 4 °C for 24 h. The physical properties were observed at the initial stage, during, and after 4 cycles.

The long-term stability test was performed by storing the gel cream at 3 different temperatures: ambient temperature, 4 °C in the refrigerator, and 45 °C in a hot air oven for 3 months. The physical properties were observed at the initial point and, subsequently, at two-week intervals.

#### 2.12.2. Stability Evaluation

The color, viscosity, and pH value of the gel creams were evaluated. The color of gel cream containing VVP was measured using a chroma meter (Chroma meter CR-400, Konica Minolta, Inc., Tokyo, Japan). The lightness (L*), redness–greenness (a*), and yellowness–blueness (b*) were measured. The viscosity was measured using a viscometer (RVDV2T extra, Brookfield, AZ, USA). The viscosity was expressed in centipoise (cps) units. The pH was measured by using a pH meter (pH-200L, NeoMet).

### 2.13. Determination of Sun Protection Factor

The Sun Protection Factor (SPF) of gel cream containing VVP was measured by using SPF 290S Analyzer (Solar Light Comp.) with Transpore Tape (ISO 24443 method). From each sample plate, the instrument randomly estimated SPF values from 5 areas and reported the SPF values as an average number from those 5 estimated values. The tests were done in triplicate on each testing and the results were expressed in terms of SPF mean and UVA/UVB ratio.

### 2.14. Irritation Test Measurement

The irritation test for gel cream containing VVP was performed using a single patch test in 20 healthy volunteers with no historical skin allergy. The patch test was carried out to determine the risk of the formulations to induce irritation to skin on short-term use. Gel cream base formula and gel cream containing VVP were placed in a chamber on the skin of the volar surface forearm, covered with an occlusive patch, and left for 24 h. The skin responses were then evaluated for irritation assessment 30 min after removal of the patch. The deionized water was used as negative control, and 1.0% sodium lauryl sulfate (SLS) was used as positive control. The skin was observed and scored for irritation from 0 (no evidence of erythema), 0.5 (minimal or doubtful erythema), 1 (slight redness, spotty and diffuse), 2 (moderate, uniform redness), 3 (strong, uniform redness), and 4 (fiery redness). All the tests performed on humans were approved by the Ethical Review Committee, Mae Fah Luang University, Thailand (EC 20128-17).

### 2.15. In Vivo Efficacy Test on Gel Cream Containing VVP

The efficacy tests for skin moisturizing using a Corneometer, melanin content using a Mexameter, elasticity and firmness using a Cutometer, and wrinkle and skin surface using a Visioscan, were performed. The 20 healthy volunteers were 14 women and 6 men aged between 23 and 45 years old. The clinical test was performed by a single blind trial. The 20 volunteers had twice daily applications of gel cream base and gel cream containing VVP on the left and right forearm every morning and evening after showering for 8 weeks. The skin assessment was carried out at week 0, 2, 4, 6, and 8. The environmental temperature and relative humidity for skin assessment were kept constant at 20 ± 2 °C and 45 ± 5%, respectively.

### 2.16. Statistical Analysis

All experiments were performed in triplicate (*n* = 3). The measured values of each analysis were analyzed using SPSS version 20. *p* values < 0.05 were regarded as statistically significant.

## 3. Results

### 3.1. Chemical Composition

**Figure 1:**
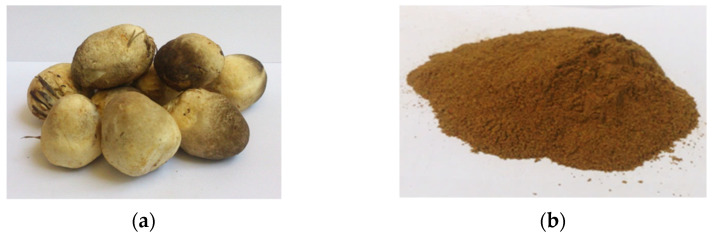
*V. volvacea*; (**a**) fresh fruiting body and (**b**) dried powder.

The dried *V. volvacea* powder appeared dark brown in color as shown in Figure 1. The chemical compositions from proximate analysis were expressed on a dried weight basis as demonstrated in Table 2. The moisture content was 8.15 ± 0.28. The carbohydrate content obtained from *V. volvacea* was 43.16 ± 0.38%, protein content was 19.40 ± 0.29%, crude fat content was 2.49 ± 0.18, ash content was 11.71 ± 0.28, and fiber content was 15.10 ± 0.16.

### 3.2. Extraction Yield

As shown in Table 3, the HS gave the highest extraction yield at 15.58 ± 0.96% which was followed by the MA and UA (11.05 ± 1.47 and 9.06 ± 0.34), respectively. The VVP yields demonstrate a significantly higher yield from HS, whereas a non-significant difference was found between UA and MA yields.

In a previous study on HS, Chen et al. [28] reported purslane polysaccharide extraction using water extraction at a solid–liquid ratio of 1:12 at 90 °C for 2 h in which a yield of 12.98% was obtained. Zhang et al. [29] reported an extraction yield of *Flammulina velutipes* polysaccharide of 9.68% by water extraction under the condition of temperature at 95 °C for 1 h with a solid–liquid ratio of 1:40. In 2015, Smolskaite et al. [30] demonstrated the extraction yields from eight different mushroom species. The highest extraction yield was *Agaricus bisporus* (17.52%) and the lowest extraction yield was *Pleurotus ostreatus* (4.32%) by water extraction under 5 h extraction periods. Consequently, this experiment shows that the conditions of temperature at 95 °C for 5 h with a solid–liquid ratio of 1:100 possess a similar extraction yield to those reported in the aforementioned experiments. For the MA result in Table 3, a similar extraction yield of 11.57% was obtained from purslane polysaccharide under the conditions of 15 min extraction, 540 Watt with a solid–liquid ratio of 1:35 [31]. Zhao et al. [32] reported the extraction yield of polysaccharide from the *Fomitopsis ulmaria* mushroom to be 8.36% under the condition 2.5 min, 400 watt with a solid–liquid ratio of 1:40. The high power of microwave extraction can cause selective migration of the target compounds from the material to the surroundings at a more rapid rate, and degradation occurred in the structure of the polysaccharides during the microwave heating process [33]. According to the above reports, it was concluded that microwave power is an important influencing factor on extraction yield. Regarding UA, Qu et al. [34] reported the extraction yield for *Ziziphus jujuba* Mill polysaccharide to be 4.47% under the conditions of temperature at 55 °C for 15 min, 120 watt with a solid–liquid ratio of 1:20. A yield of 6.02% from *Agaricus bisporus* polysaccharide was derived under the conditions of temperature at 70 °C for 15 min, 230 Watt with a solid–liquid ratio of 1:30 [35]. The results in Table 3 show that a higher yield of 9.06% was observed in this study. With an increase in ultrasonic power (UA), the ultrasonic effects on the cell wall also increase. In addition, when the power is too strong, UA can lead to liquid flowing too fast. This implies that the residence time of the material is reduced, and the extraction yield is also decreased by strong power [33].

### 3.3. Solubility of VVP

The solubility of VVP is 12 mg/mL or 1.20% *w*/*v* so it is considered a highly soluble polysaccharide. Water soluble polymers have a wide range of industrial applications, such as in food, pharmaceuticals, paint, textiles, paper, construction, adhesives, coating, and water treatment. In cosmetic applications, polysaccharides are widely used as a natural thickening agent in various cosmetic dosage forms, a film-forming agent in color and make-up products, and a heat-protecting agent in hair-styling products. They are also extensively used as a natural moisturizing agent to hydrate facial and body skin. Most polysaccharides contain glycosyl units that on average have three hydroxyl groups. Each hydroxyl group on a polysaccharide has the possibility of hydrogen bonding to one or more water molecules. Also, the ring oxygen atom and the glycosidic oxygen atom connecting one sugar ring to another can form hydrogen bonds with water. In aqueous systems, polysaccharide particles can take up water, swell, and usually undergo partial or complete dissolution. Polysaccharides possess a strong affinity for water and readily hydrate when water is available [36]. Furthermore, β-glucans are non-starch polysaccharides which present either in their water-soluble or insoluble form. The biological activity of the water-soluble form was much greater in humans and animals and had a more pronounced effect on their immune systems [37].

### 3.4. Polysaccharide Content

The polysaccharide contents in the extracts were determined by the phenol–sulfuric method and their results are presented in Table 3. The polysaccharide contents were statistically non-significantly different among the three extraction methods. Kozarski et al. [38] determined the polysaccharide contents of extracts from hot water extraction from various medicinal mushrooms: *Ganoderma applanatum*, *G. lucidum*, *Lentinus edodes* and *Trametes versicolor*. Total polysaccharide contents ranged between 0.57 and 0.84 g/g dried weight extract with the lowest and highest values observed from *G. lucidum* and *T. versicolor*, respectively; however, the different types of mushrooms investigated might play a major role in the large difference in polysaccharide contents. Differences in some extraction conditions, for example extraction time, temperature, and the power of equipment used, may also affect the yield.

### 3.5. Beta-Glucan Content

The β-glucan content in the VVP was determined as it is a major polysaccharide in this mushroom. As shown in Table 3, the HS sample shows the significantly highest β-glucan content (18.80 ± 0.81%, *w*/*w*) followed by the UA sample of 14.29 ± 0.73% *w*/*w*. The lowest yield of β-glucan was obtained from MA which may be because the high power of microwave extraction leads to structural degradation occurring during the microwave-heating process [37]. The differences in the β-glucan amounts in each mushroom might be due to the difference in the water solubility-related β-glucan structure. It was reported that for the backbone chain of β-(1,3)-linked-D-glucose residues, one out of five or six found in *V. volvacea* are substituted at O-6 with single glucosyl or β-(1,6)-linked diglucosyl groups, whereas, the β-1,3-branch containing mannose, glucose, xylose, and glucuronic acid units was found in *A. auricular* [13,18].

Beta-glucans were reported to reduce infections and enhance the strength of tissue in wound healing both in animals and humans [6,7]. Moreover, β-glucan was applied in the cosmetics field due to its anti-wrinkle, anti-inflammatory, antioxidant, and moisturizing effects [39]. Wu et al. [40] reported the applications in cosmetics of β-glucan as increasing skin moisturization, increasing collagen production, and reducing wrinkles.

### 3.6. VVP Antioxidant Capacity

**Figure 2:**
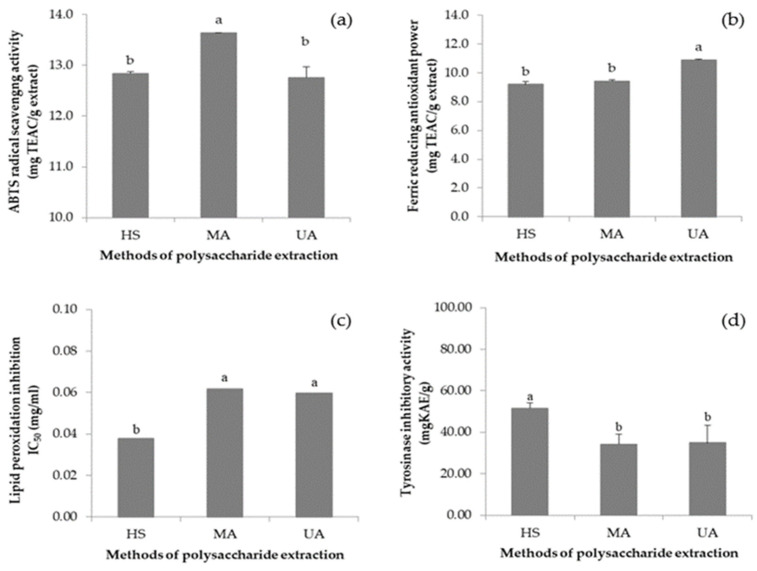
Antioxidant activities of (**a**) ABTS-radical scavenging activity; (**b**) FRAP; (**c**) lipid peroxidation inhibitory; and (**d**) anti-tyrosinase activities of the VVP by hot water shaking (HS), microwave-assisted (MA), and ultrasonic-assisted (UA). Different lowercase indicates a significant difference (*p* < 0.05).

Antioxidant capacity was determined using three assays, namely the ABTS radical scavenging capacity assay, the ferric-reducing antioxidant power (FRAP) assay, and the lipid peroxidation assay. The results of ABTS radical scavenging in VVP are shown in Figure 2a. The VVP possess an ABTS-radical scavenging activity of 12.84 ± 0.04 mg TEAC/g (HS), 13.64 ± 0.00 mg TEAC/g (MA), and 12.76 ± 0.21 mg TEAC/g (UA). For the FRAP assays, as shown in Figure 2b, the VVP possess a reducing power of 9.19 ± 0.18 mg TEAC/g (HS), 9.42 ± 0.10 mg TEAC/g (MA), and 10.89 ± 0.04 mg TEAC/g (UA). The results of lipid peroxidation inhibition by VVP are shown in Figure 2c: the VVP exhibits the highest efficacy with IC_50_ of 0.0378 mg/mL from HS extraction, followed by 0.0597 mg/mL and 0.0617 mg/mL from UA and MA, respectively. It is noticed that all VVPs demonstrate higher inhibitory activity when compared with BHT (IC_50_ = 0.1609 mg/mL).

Smolskaite et al. [30] studied the antioxidant capacity of different mushroom species and the highest ABTS radical scavenging was found in *Inonotus hispidus* polysaccharides extracted by hot water shaking (41.299 mg TEAC/g). A previous report by Zhang et al. [41] studied *Armillaria mellea*, a traditional Chinese mushroom, in which polysaccharides extracted by HS were investigated for antioxidant activities. The antioxidant activities were measured by using ABTS and FRAP assays. The IC_50_ values of *A. mellea* mycelium polysaccharide were 1.35 mg/mL for the ABTS assay and 0.552 mg/mL for the FRAP assay. The symbionts of lichen species *Arthothelium awasthii*, *Heterodermia podocarpa*, and *Parmotrema tinctorum* were cultured and their polysaccharides were extracted with methanol. The inhibition of lipid peroxidation was expressed in IC_50_ which was 15.7 μg/mL for *A. awasthii*, 12.68 μg/mL for *H. podocarpa*, and 11.47 μg/mL for *P. tinctorum*. The tested extracts were also more effective than the standard antioxidant trolox (IC_50_ 16.13 μg/mL) [42].

### 3.7. VVP Tyrosinase Inhibitory Activity

In this study, L-Dopa was used as the substrate for determining tyrosinase inhibitory activity. The results are given in Figure 2d. The VVP HS demonstrates the highest tyrosinase inhibitory activity (51.46 mg KAE/g), whereas the extracts from MA and UA possess lower inhibitory activity with no statistical difference between them (34.88 ± 8.41 and 34.17 ± 4.95 mg KAE/g, respectively).

The tyrosinase inhibitory activities of *P. ostreatus* fruiting body polysaccharides extracted with acetone (25 °C for 24 h), methanol (25 °C for 24 h), and hot water (100 °C for 3 h) were evaluated [43]. The results were 11.37–52.50%, 11.36–59.56%, and 9.40–49.60%, respectively, when compared with standard ascorbic acid. According to Verma et al. [42], the polysaccharides from lichen presented higher tyrosinase inhibitory activity than 1.0% (*w*/*v*) of standard kojic acid (IC_50_ = 17.63 μg/mL); for example, 8.71 μg/mL for *A. awasthii*, 14.55 μg/mL for *H. podocarpa*, and 12.44 μg/mL for *P. tinctorum*. Hence, the polysaccharides displaying tyrosinase inhibitory activity could have potential use as whitening agents in cosmetic and topical treatment products.

### 3.8. VVP Elastase Inhibitory Activity

In this study, epigallocatechin gallate (EGCG) was used as the standard substance for determining elastase inhibitory activities. The highest elastase inhibitory activity of the VVP was obtained from HS (604.21 ± 73.66 mg EGCG/g), followed by UA (107.65 ± 3.47 EGCG/g). Anti-elastase activity was not detected in the MA polysaccharides. This could be explained by the fact that HS is a mild extraction condition, while MA and UA extraction might rupture polysaccharide molecules by rotation, vibration, and collision during extraction [44] resulting in an improper structure arrangement, thereby preventing effective binding with the elastase enzyme.

The fruiting body of *Dictyophora indusiata* (veiled lady mushroom) was extracted by methanol for study of its anti-cholinesterase, skin anti-wrinkle, and melanogenesis inhibitory activity [45]. The collagenase and elastase inhibitory activities of the extract were comparable with epigallocatechin gallate (EGCG) as the positive control, which suggested it could be a good candidate for natural anti-cholinesterase and as a skin care agent [45]. It was found that extract of the mycelium of *Tricholoma matsutake* (Pine mushroom) significantly decreased elastase activity in a dose-dependent manner and reduced the levels of MMPs [46]. The inhibitory effect of *T. matsutake* mycelium extract (81.4 ± 3.92%) toward human fibroblast elastase was observed at a concentration of 100 μg/mL. The extract was proven to be an effective biomaterial for anti-wrinkle treatment in cosmetics products [46].

### 3.9. VVP UV Absorption Scanning 

**Figure 3:**
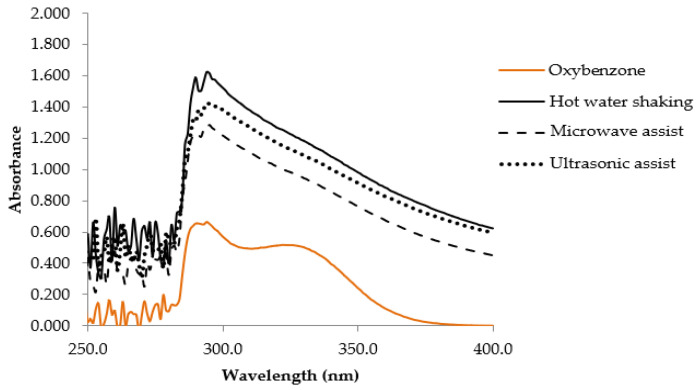
UV absorption scanning of VVP from different extraction methods.

The scanning profiles of VVP (1.0 mg/mL) are shown in Figure 3. The interesting absorbability ranges between 280 and 300 nm and belongs to the UVB region, and the maximum absorbance of 294 nm was found in the HS VVP. When compared with the oxybenzone standard (0.01 mg/mL), a maximum wavelength of approximately 294 nm and a high absorption capacity of around 328 nm were observed. Similar UVB absorption with oxybenzone suggests that the VVP could probably be used as a sun-screening agent. Oxybenzone is a chemical sunscreen which is cosmetically attractive due to being invisible on the skin’s surface; however, it can cause contact dermatitis and photosensitivity reactions [47]. The VVP might be interesting in terms of its use as an alternative natural sunscreen and its potential lower health risk.

### 3.10. VVP Cell Toxicity 

The VVP extracted by hot water shaking was subjected to the cytotoxicity test against human dermal skin fibroblasts. The results demonstrate non-cytotoxicity in that more than 98% of the cells were still alive when a VVP concentration higher than 200 µg/mL was used. This indicates that VVP is safe for use in topical products. These results accord with the fact that *V. volvacea* has been consumed by humans for a long time and is considered to be a medicinal food.

### 3.11. Development of Facial Gel Cream Containing VVP

**Figure 4:**
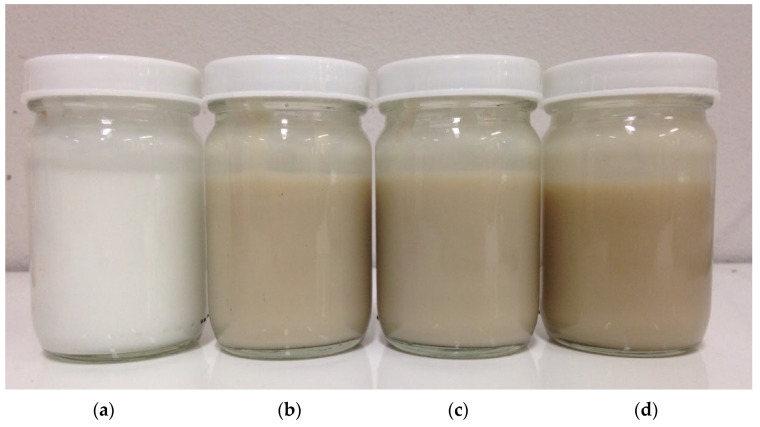
Appearance of (**a**) gel cream base; and gel cream containing VVP at (**b**) 0.20%; (**c**) 0.50%; and (**d**) 1.00% *w*/*w*.

The gel cream base was prepared in the form of oil-in-water emulsion providing a light-texture watery feeling, but absorbing well into the skin. The VVP extracted by HS was selected for use as an active ingredient due to its high antioxidant activity, tyrosinase inhibitory activity, and elastase inhibitory activity. The viscosity, pH value, and the color of the product were evaluated for their stability during and after acceleration testing and long-term storage. The appearance of the gel cream containing three different concentrations of VVP is shown in Figure 4. The higher amount of VVP in the formula resulted in a darker color due to the color of the raw material; however, all formula colors were acceptable in terms of a natural extract-containing product.

#### 3.11.1. Heating–Cooling Stability

**Figure 5:**
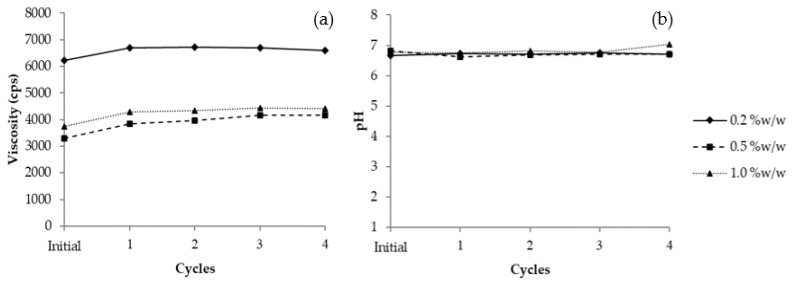
Profiles of VVP gel cream after treatment with 4 cycles of heating–cooling test: (**a**) Viscosity and (**b**) pH. The viscosity test used Spindle No. 5, 30 rpm at 25 °C.

A high and low temperature cycle test is a widely used method to assess the stability of cosmetics. As the results show in Figure 5a, the 0.2% *w*/*w* VVP had a higher viscosity (6227 cps) than the 0.5% *w*/*w* (3307 cps) and 1.0% *w*/*w* (3733 cps). Their viscosities increased slightly after treatment with four cycles of heating–cooling; however, the ranges of viscosity change in all the formulations were acceptable in terms of stability. The pH value of the 0.2% *w*/*w* VVP formulation was 6.67–6.76; this was similar to that of the 0.5% *w*/*w*, which was between 6.70 and 6.81. The pH of the 1.0% *w*/*w* formulation was slightly higher at 6.76–7.03 after four cycles of the heating–cooling process, as shown in Figure 5b. Based on the results for pH stability, it is assumed that the products containing 0.20–1.00% *w*/*w* of VVP are stable after heating–cooling acceleration.

**Figure 6:**
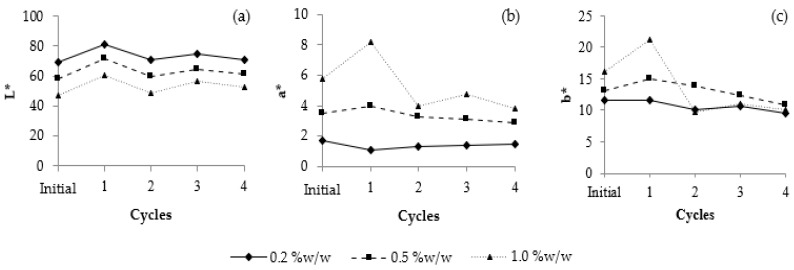
Color profile of polysaccharide formulation after treatment with 4 cycles of heating–cooling: (**a**) L* values; (**b**) a* values; and (**c**) b* values.

Color change was monitored over the duration of the study. The results are shown in Figure 6: at the initial stage, the 0.20% and 0.50% VVP formula were light brown in color (L* = 68.94; a* = 1.73; b* = 11.58 and L* = 57.82; a* = 3.49; b* = 13.18, respectively); the 1.00% VVP formula was dark brown in color (L* = 47.19; a* = 5.79; b* = 16.09). The colors of the gel cream presented depend on the amount of polysaccharide content. During the heating–cooling cycles, all formulations showed slightly increased lightness, with a decrease in redness and yellowness. Although some changes in the pH, viscosity, and color of the 0.2% VVP formula were observed after the acceleration test, these changes were acceptable and could not be distinguished by eye perception; therefore, 0.20% VVP gel cream was selected for the long-term stability study.

#### 3.11.2. Long-Term Stability

**Figure 7:**
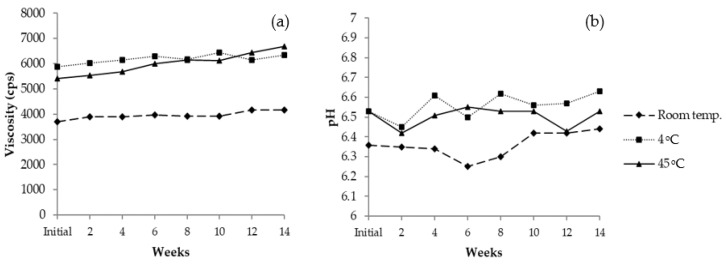
Profiles of polysaccharide formulation after being stored under three conditions for 3 months: (**a**) Viscosity and (**b**) pH.

**Figure 8:**
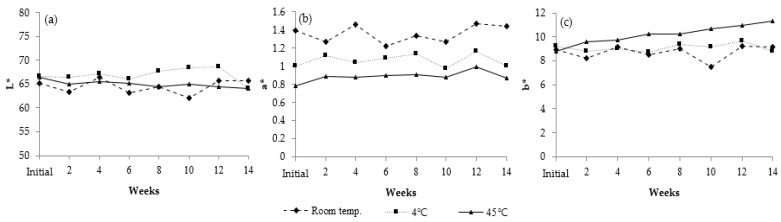
Color of polysaccharide formulation after being stored under three conditions for 3 months: (**a**) L* values; (**b**) a* values; and (**c**) b* values.

The 0.2% VVP gel cream was selected for long-term stability evaluation at room temperature, 4 °C and 45 °C. The viscosity of the gel cream containing 0.2% VVP increased slightly after three months, ranging from 3693 to 4160 cps at room temperature, 5893 to 6347 cps at 4 °C, and 5413 to 6693 cps at 45 °C (Figure 7a). The pH values of VVP gel cream ranged between 6.25 and 6.44, implying the product had good stability under the three different conditions (Figure 7b). The results of color evaluation during storage are illustrated in Figure 8. The redness (+a*) and the yellowness (+b*) of the formulation increased slightly at room temperature and 45 °C so it could be assumed that high temperature resulted in deep yellowness. Although some changes in pH, viscosity, and color were observed after the acceleration test, these changes were acceptable and could not be distinguished by eye perception. Therefore, the gel cream containing 0.2% VVP was further studied for irritation and in vivo efficacy tests.

### 3.12. Irritation Test

The twenty healthy volunteers, fourteen women and six men, aged between 23 and 45 years old had gel cream containing VVP applied. Deionized water was used as the negative control, and 1.0% sodium lauryl sulfate (SLS) was the positive control.

The results from the patch test revealed that all volunteers experienced neither allergy nor irritation (irritation score = 0) after the application of the gel cream base and gel cream containing VVP. This indicates the non or very low risk of the formulations causing skin irritation. In contrast, volunteers showed slight to moderate redness (score = 1–2) after 30 minutes of the 1.0% (SLS) application.

### 3.13. Sun Protection Factor (SPF)

In vitro SPF of the base cream formula and the cream containing 0.2% VVP were studied, and the results are shown in Table 4. The SPF of the base formula is 0.99, while that of the gel cream containing VVP is 1.02. The critical wavelength was increased when VVP was added; however, it could not be claimed to be a broad spectrum due to the critical wavelength being less than 370 [48]. Even though the VVP illustrate UVB absorption, as shown in Figure 3, the gel cream containing VVP shows a lower SPF value, implying it provides less UVB protection to skin. Still, previous studies reported that polysaccharides have a potential photoprotective capacity against UV rays [49]. The lower UV protection observed from the VVP gel cream in this study might result from the low concentrations of the VVP. Likewise, a high SPF value is usually obtained from formulations which include substantial amounts of physical sunscreen. Including a higher amount of VVP combined with a physical sunscreen in the formulation would probably enhance the SPF value.

### 3.14. Anti-Wrinkle, Moisturization and Whitening Efficiencies of Gel Cream Containing VVP

**Figure 9:**
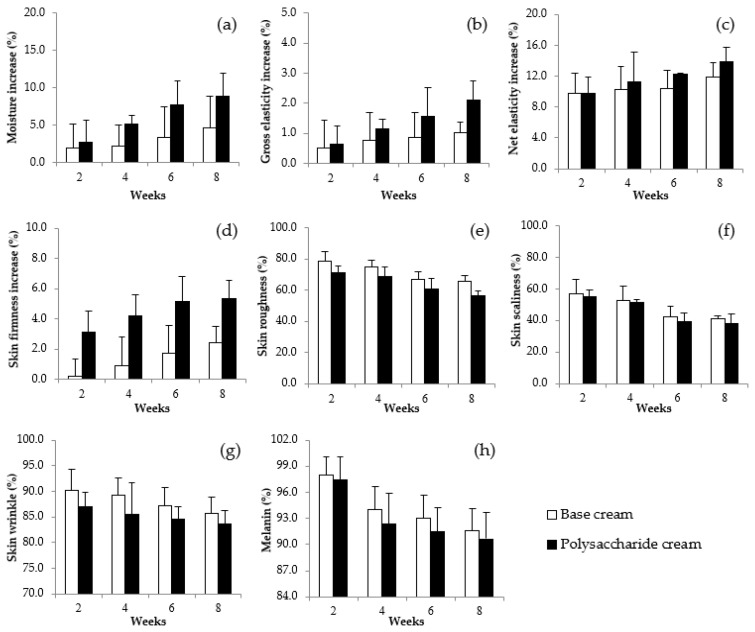
Skin measurement of 20 healthy volunteers after application of the base formula compared to the facial moisturizing gel cream containing mushroom: (**a**) average moisture increases; (**b**) average gross elasticity increases (%); (**c**) average net elasticity increase; (**d**) average skin firmness increase; (**e**) average skin roughness; (**f**) average skin scaliness; (**g**) average skin wrinkle; and (**h**) average skin melanin content.

A clinical study to measure the efficacy of the VVP was performed on twenty volunteers. The moisture content in skin with gel cream containing VVP (Figure 9a) rose continuously to approximately 8.87% when compared with before application, while moisture content in the skin with base formula was 4.61% when compared with before application. The higher moisture content obtained from the gel cream containing VVP indicates that the polysaccharide from *V. volvacea* greatly enhances skin moisturization on human skin. A previous study reported that *Tremella* polysaccharides (0.05%) showed superior moisture retention capacity than did 0.02% hyaluronic acid [9]. Polysaccharides contain a number of hydroxyl groups in their structures which are able to bind with water molecules and subsequently provide more hydration to the skin.

Skin elasticity was evaluated through gross elasticity and net elasticity parameters using a Cutometer. Gross elasticity (Figure 9b) rose continuously to approximately 2.11% when compared with before application, while the gel cream base increased it to approximately 1.02%. The net elasticity (Figure 9c) from the VVP gel cream was elevated to approximately 13.88%, while that of the base formula was 11.88% when compared with before application. The skin elasticity values of gel cream containing VVP were higher than those of the gel cream base and it possessed good skin improvement. Skin firmness (Figure 9d) data show that the VVP gel cream exhibits a higher value with a 5.32% increase, while the base formula provides a lower increment in skin firmness at 2.44%. The results appear to show that the VVP gel cream provides higher anti-wrinkle efficacy than does the gel cream base.

The volunteers’ skin surface was also evaluated through skin roughness, skin scaliness and skin wrinkle parameters using a Visioscan. After application of the gel cream containing 0.2% VVP for eight weeks, skin roughness (Figure 9e) decreased continuously reaching a total decrease of approximately 43.6% when compared with before application, whereas the base formula appeared to decrease to approximately 34.5%. It can be seen that the VVP gel cream indicates a greater reduction in skin roughness than does the gel cream base. Skin scaliness (Figure 9f) also decreased approximately 61.78% when compared with that of the base formula which exhibited a value of 59.08% in scaliness decline. The volunteers showed a decline of approximately 16.28% in their skin wrinkles at week 8 (Figure 9g). This wrinkle decrease is higher than that of the gel cream base which revealed a value of 14.28%. These parameters indicate good skin improvement, especially for skin wrinkles. It could be assumed that gel cream containing VVP could reduce skin wrinkles and act as an anti-wrinkle cream.

The melanin contents of volunteer skin (Figure 9h) with VVP gel cream decreased continuously, achieving a total decrease of approximately 9.4% at week 8 compared to an approximate 1% melanin decrease in skin with the base formula. The reduction in melanin content suggests that the gel cream containing VVP also provides a whitening effect to the skin. This is similar to a previous investigation in which the hot water extract of a *Tremella fuciformis* mushroom, without the addition of a chemical reagent, was found to have the novel effect of inhibiting melanin formation and lightening the spots on the skin when applied [50].

The results of this study are the first-time report on an efficacy test on human skin for a cosmetic product containing VVP. The results illustrate that VVP is appropriate for improving skin esthetic problems including dryness, darkness, and wrinkles. Hence, a gel cream containing VVP can be considered as a multi-functional cosmetic for moisturizing, anti-aging, and whitening. This agrees with a previous review that revealed the uses of mushroom polysaccharides as active ingredients in commercial cosmetic preparations for many purposes, such as moisturizing, anti-aging, and skin whitening [40].

## 4. Conclusions

The *V. volvacea* polysaccharides extracted by the hot water shaking (HS) method demonstrated a significant highest percent yield and β-glucan contents. Lipid peroxidation, tyrosinase, and elastase inhibitory activities were also significantly highest in the polysaccharide extracted by HS. The gel cream containing VVP had good stability after testing with four cycles of heating–cooling treatment and after storage at room temperature, 4 °C, and 45 °C for 3 months. The efficacy test on twenty volunteers showed the enhancing effects of the VVP gel cream in skin moisturizing, skin elasticity, and skin firmness. Moreover, it demonstrated a reduction in skin roughness, skin scaliness, and skin melanin content. This study is the first report of efficacy testing on human skin of a cosmetic product containing VVP. It can be concluded that the polysaccharides from *V. volvacea* possess high potential as an active cosmetic ingredient.

## Figures and Tables

**Table 1:** Base formula and gel cream containing VVP.

Part	Ingredient	Formula			
		Base	0.2%	0.5%	1.0%
A	Deionized water	85.25	85.05	84.75	84.25
	Sodium Acrylates/Beheneth-25 Methacrylate Crosspolymer (and) Hydrogenated Polydecene (and) Lauryl Glucoside	2.00	2.00	2.00	2.00
	Hydroxyethyl Acrylate/Sodium Acryloyldimethyl Taurate Copolymer (and) squalane (and) polysorbate 60	1.00	1.00	1.00	1.00
B	Deionized water	10.00	10.00	10.00	10.00
	Citric acid	0.05	0.05	0.05	0.05
C	Isopropyl myristate	1.50	1.50	1.50	1.50
D	*V. Volvacea* polysaccharide (VVP)	-	0.20	0.50	1.00
E	Phenoxyethanol	0.20	0.20	0.20	0.20

**Table 2:** Proximate analysis of *V. volvacea* mushroom.

Composition	Percentage (% Dried Weight Basis)
Crude fat	2.49 ± 0.18
Protein	19.40 ± 0.29
Carbohydrate	43.16 ± 0.38
Fiber	15.10 ± 0.16
Ash	11.71 ± 0.28
Moisture	8.15 ± 0.28

**Table 3:** Extraction yields, polysaccharide, and beta-glucan contents VVP.

Analyses	Hot Water Shaking (HS)	Microwave Assisted (MA)	Ultrasonic Assisted (UA)
Yield (%)	15.58 ± 0.96 ^a^	11.05 ± 1.47 ^b^	9.06 ± 0.34 ^b^
Total polysaccharide (g GE/g)	0.46 ± 0.02 ^a^	0.58 ± 0.06 ^a^	0.59 ± 0.13 ^a^
Beta-glucans (%*w*/*w*)	18.80 ± 0.81 ^a^	9.75 ± 0.53 ^b^	14.29 ± 0.73 ^c^

Different lowercase indicates a significant difference (*p* < 0.05) in the same roll.

**Table 4:** Sun Protection Factor (SPF) in gel cream base and gel cream containing VVP.

Parameters	Sample	
	Gel Cream Base	Gel Cream with 0.2% VVP
SPF mean	0.99	1.02
Critical wavelength	54.8	212.9
UVA/UVB ratio	0.015	−0.463
Boots Star Rating	Not detected	Not detected
